# Prenatal chromosomal microarray analysis in a large Chinese cohort of fetuses with congenital heart defects: a single center study

**DOI:** 10.1186/s13023-024-03317-4

**Published:** 2024-08-22

**Authors:** Qing Lu, Laipeng Luo, Baitao Zeng, Haiyan Luo, Xianjin Wang, Lijuan Qiu, Yan Yang, Chuanxin Feng, Jihui Zhou, Yanling Hu, Tingting Huang, Pengpeng Ma, Ting Huang, Kang Xie, Huizhen Yuan, Shuhui Huang, Bicheng Yang, Yongyi Zou, Yanqiu Liu

**Affiliations:** 1 Medical Genetic Center, Jiangxi Maternal and Child Health Hospital, No. 318, Bayi Avenue, Nanchang, China; 2 Department of Ultrasound, Jiangxi Maternal and Child Health Hospital, No. 318, Bayi Avenue, Nanchang, China; 3 Jiangxi Key Laboratory of Birth Defect Prevention and Control, No. 318, Bayi Avenue, Nanchang, China

**Keywords:** Congenital heart defects, Chromosomal abnormalities, Copy number variations, Chromosomal microarray analysis, Prenatal diagnosis

## Abstract

**Background and objectives:**

Congenital heart defect (CHD) is one of the most common birth defects. The aim of this cohort study was to evaluate the prevalence of chromosomal abnormalities and the clinical utility of chromosomal microarray analysis (CMA) in fetuses with different types of CHD, aiming to assist genetic counseling and clinical decision-making.

**Methods:**

In this study, 642 fetuses with CHD were enrolled from a single center over a six-year period (2017–2022). Both conventional karyotyping and CMA were performed simultaneously on these fetuses.

**Results:**

The diagnostic yield of CMA in fetuses with CHD in our study was 15.3% (98/642). Our findings revealed a significant increase in the diagnostic yield of CMA compared to karyotyping in fetuses with CHD. Among CHD subgroups, the diagnostic yields were high in complex CHD (34.9%), conotruncal defects (28.6%), right ventricular outflow tract obstructive defects (RVOTO) (25.9%), atrioventricular septal defects (AVSD) (25.0%) and left ventricular outflow tract obstructive defects (LVOTO) (24.1%), while those in other CHD (10.6%) and septal defects (10.9%) were relatively low. The overall detection rate of clinically significant chromosomal abnormalities was significantly higher in the non-isolated CHD group compared to the isolated CHD group (33.1% vs. 9.9%, *P* < 0.0001). Interestingly, numerical chromosomal abnormalities were more likely to occur in the non-isolated CHD group than in the isolated CHD group (20.3% vs. 2.0%, *P* < 0.0001). The rate of termination of pregnancy (TOP)/Still birth in the non-isolated CHD group was significantly higher than that in the isolated CHD group (40.5% vs. 20.6%, *P* < 0.0001). Compared to the isolated CHD group, the detection rate of clinically significant chromosomal abnormalities was significantly higher in the group of CHD with soft markers (35.6% vs. 9.9%, *P* < 0.0001) and in the group of CHD with additional structural anomalies (36.1% vs. 9.9%, *P* < 0.0001).

**Conclusions:**

CMA is a reliable and high-resolution technique that should be recommended as the front-line test for prenatal diagnosis of fetuses with CHD. The prevalence of chromosomal abnormalities varies greatly among different subgroups of CHD, and special attention should be given to prenatal non-isolated cases of CHD, especially those accompanied by additional structural anomalies or soft markers.

**Supplementary Information:**

The online version contains supplementary material available at 10.1186/s13023-024-03317-4.

## Background

Congenital heart disease (CHD) is a structural anomaly of the heart and/or great vessels that arises due to disruptions in the normal developmental program of the cardiac system. CHD is one of the most common birth defects, affecting 8–9 babies out of every 1,000 live births and occurring in 10% of spontaneous miscarriages [1, 2]. It is estimated that over 130,000 babies are born with CHD each year in China, with annual treatment costs reaching up to $1.6 billion [3]. The etiology of CHD is complex, involving both genetic and non-genetic factors [4]. Despite significant advancements in the diagnosis and treatment of CHD, our understanding of its underlying causes has been limited until recently. Approximately 20% of cases with CHD have identifiable etiologies, while the majority remain sporadic with unknown origins. Genetic factors include chromosomal abnormalities, genetic syndromes, and single-gene disorders; whereas potential non-genetic factors comprise environmental teratogens, maternal exposures, and infectious agents [5–7].

With significant advancements in ultrasound technologies and improvements in equipment capabilities, the prenatal detection of cardiovascular malformations through ultrasound has witnessed a remarkable increase. Chromosomal abnormalities are recognized as a predominant genetic factor contributing to CHD, with their identification reported in over 20% of fetuses affected by this condition [8, 9]. Aneuploidies represent the earliest and most prevalent chromosomal abnormalities associated with cases of CHD, with trisomy 21 being the most frequently observed, followed by trisomy 18 and trisomy 13 [10]. Additionally, copy number variations (CNVs) are recognized as crucial chromosomal abnormalities in CHD cases. Previous studies have identified specific CNVs associated with CHD, including 22q11.2 deletion, 1q21.1 deletion and duplication, 1p36 deletion, 3p25.1 deletion, 7q11 deletion, 8p23 deletion, 11q24-25 deletion, 15q11.2 deletion and 16p13.1 duplication [11]. These CNVs typically encompass genes associated with CHD or genes known to play a crucial role in cardiac development. For example, the 22q11.2 deletion alters the dosage of a gene, *TBX1*, a T-box transcription factor facilitates cellular proliferation in the secondary heart field, which gives rise to the development of the outflow tract and right ventricle [2]. However, it is widely acknowledged that fetuses diagnosed with both CHD and chromosomal anomalies generally have a poor prognosis [12, 13]. Therefore, we believe that the genetic diagnosis of fetuses with CHD plays a pivotal role in prenatal counseling and prognosis evaluation, making it highly recommended.

Although karyotyping and fluorescence in situ hybridization (FISH) have been used for detecting chromosomal abnormalities in prenatal CHD cases over the past few decades, these approaches possess certain significant limitations. For example, karyotyping is time-consuming and has low resolution, while FISH has limited coverage; however, chromosomal microarray analysis (CMA), which is designed to identify microscopic and submicroscopic chromosomal abnormalities, has recently been widely used in the field of prenatal diagnosis. Therefore, we conducted a cohort study involving 642 fetuses with CHD at a single center to assess both the prevalence of chromosomal abnormalities and the clinical utility of CMA as a genetic diagnostic tool for prenatal CHD cases. Additionally, we categorized these cases into different CHD subgroups to gain better insights into the diagnostic yields of chromosomal abnormalities and provide guidance for genetic counseling and clinical decision-making in prenatal CHD cases.

## Methods

### Participants

During the period from January 2017 to June 2022, pregnant women were consecutively referred to the Medical Genetic Center of Jiangxi Maternal and Child Health Hospital for invasive prenatal testing due to factors such as advanced maternal age, a high risk of prenatal screening, a family history of hereditary disease or abnormal pregnancy, and fetuses with ultrasound abnormalities. Pregnant women diagnosed with fetal CHD were recruited for this study. Fetuses with CHD were identified through routine ultrasound anatomy scans in the first or second trimesters and subsequently confirmed by echocardiography. Karyotyping and CMA, which are routine diagnostic tests for chromosomal anomalies, were performed simultaneously in all participants. Prior to invasive prenatal testing, all participants were duly informed about the benefits and potential risks associated with CMA and provided with written informed consent. All fetal specimens were amniotic fluid and collected through amniocentesis. Soft markers refer to minor and transient structural changes that may indicate potential significant risks of fetal abnormalities, yet are often inconsequential in isolation. Based on the presence or absence of extracardiac ultrasound anomalies such as additional structural anomalies, soft markers or amniotic fluid volume abnormality, all cases were divided into an isolated group and a non-isolated group. Single umbilical artery, absent or shortened nasal bones, echogenic bowels, choroid plexus cysts, mild ventriculomegaly (10–15 mm), increased nuchal translucency (≥ 3.0 mm), enlarged cisterna magna, thickened nuchal folds (≥ 6.0 mm), persistent right umbilical veins and pyelectasis were considered as soft markers in this study. Additionally, all cases were categorized into a series of CHD groups and subgroups by Botto’s method [14]. After the birth of surviving fetuses, participants were recommended to undergo a comprehensive ultrasound examination. All participants were followed up through phone interview to inquire about the outcomes of their pregnancy. This study was approved by the Medical Ethics Committee of Jiangxi Maternal and Child Health Hospital.

### CMA and karyotyping for routine prenatal diagnosis

The Affymetrix CytoScan 750 K array (Applied Biosystems, Affymetrix, Inc., Santa Clara, CA, USA) has been routinely utilized for prenatal genetic testing in our center. It encompasses a total of 750,000 probes distributed across the entire human genome. CMA was performed in all enrolled cases using the SNP-based platform of CytoScan 750 K. Fetal genomic DNA was extracted from amniotic fluid cells utilizing the commercially available QIAamp^®^ DNA Mini Kit (Qiagen, Inc., Hilden, Germany). The experimental procedures were conducted in accordance with the manufacturer’s protocols. The data was analyzed using the Affymetrix^®^ Chromosome Analysis Suite (ChAS) v4.2 Software, and the results were mapped to the Genome Reference Consortium Human Build 37 (GRCh37/hg19). After passing quality control standards, copy number variants (CNVs) were systematically evaluated by comparing them with scientific literature and consulting various public databases, including UCSC Genome Browser (http://genome.ucsc.edu/), ISCA (http://clinicalgenome.org/), Pubmed (http://www.ncbi.nlm.nih.gov/pubmed/), Database of Genomic Variants (http://dgv.tcag.ca/dgv/app/home), OMIM (http://omim.org/), DECIPHER (https://decipher.sanger.ac.uk/), and ClinGen Dosage Sensitivity Map (http://www.ncbi.nlm.nih.gov/projects/dbvar/clingen/index.shtml). According to the standards and guidelines of the American College of Medical Genetics (ACMG), CNVs were classified into five categories: pathogenic CNVs, likely pathogenic CNVs, variants of uncertain significance (VUS), likely benign CNVs, and benign CNVs. In this study, clinically significant CNVs included pathogenic and likely pathogenic CNVs; however, likely benign and benign CNVs were not reported. Both aneuploidies and clinically significant CNVs were considered as clinically significant chromosomal abnormalities. G-banding karyotyping was performed in all cases following the standard procedure [15].

### Statistical analyses

Chi-square test or Fisher exact test was used to compare the detection rate of chromosomal abnormalities in different CHD groups. *P* ≤ 0.05 was considered statistically significant.

## Results

### Characteristics of patients

From January 2017 to June 2022, a total of 16,362 fetuses underwent prenatal diagnosis at our center. Among them, 642 fetuses with CHD were included in this study, accounting for 3.9% (642/16,362) of the entire population that was diagnosed. The demographic characteristics of all enrolled cases are summarized in Table 1. The overall mean age of the pregnant women was 28.0 ± 5.0 years, and the mean gestational age at invasive testing was 24.3 ± 2.9 weeks. The overall rates of Ongoing/Live birth and termination of pregnancy (TOP)/Still birth in all fetuses with CHD were 74.8% (480/642) and 25.2% (162/642), respectively. The rate of TOP/Still birth in the non-isolated CHD group was significantly higher than that in the isolated CHD group (40.5% vs. 20.6%, *P* < 0.0001).

**Table 1:** Demographic characteristics of the 642 enrolled CHD cases

	Total (*n* = 642)	Isolated CHD group (*n* = 494)	Non-isolated CHD group (*n* = 148)
Mean maternal age (years)	28.0 ± 5.0	27.9 ± 4.8	28.3 ± 5.3
Gestational week at invasive testing (weeks)	24.3 ± 2.9	24.4 ± 2.6	23.8 ± 3.8
Pregnancy outcome			
Ongoing/Live birth, n (%)	480(74.8%)	392(79.4%)	88(59.5%)
TOP/Still birth, n (%)	162(25.2%)	102(20.6%)	60(40.5%)

CHD: Congenital heart defects; n: number of cases; TOP: termination of pregnancy

### Diagnostic yield of karyotyping vs. CMA

**Fig. 1:**
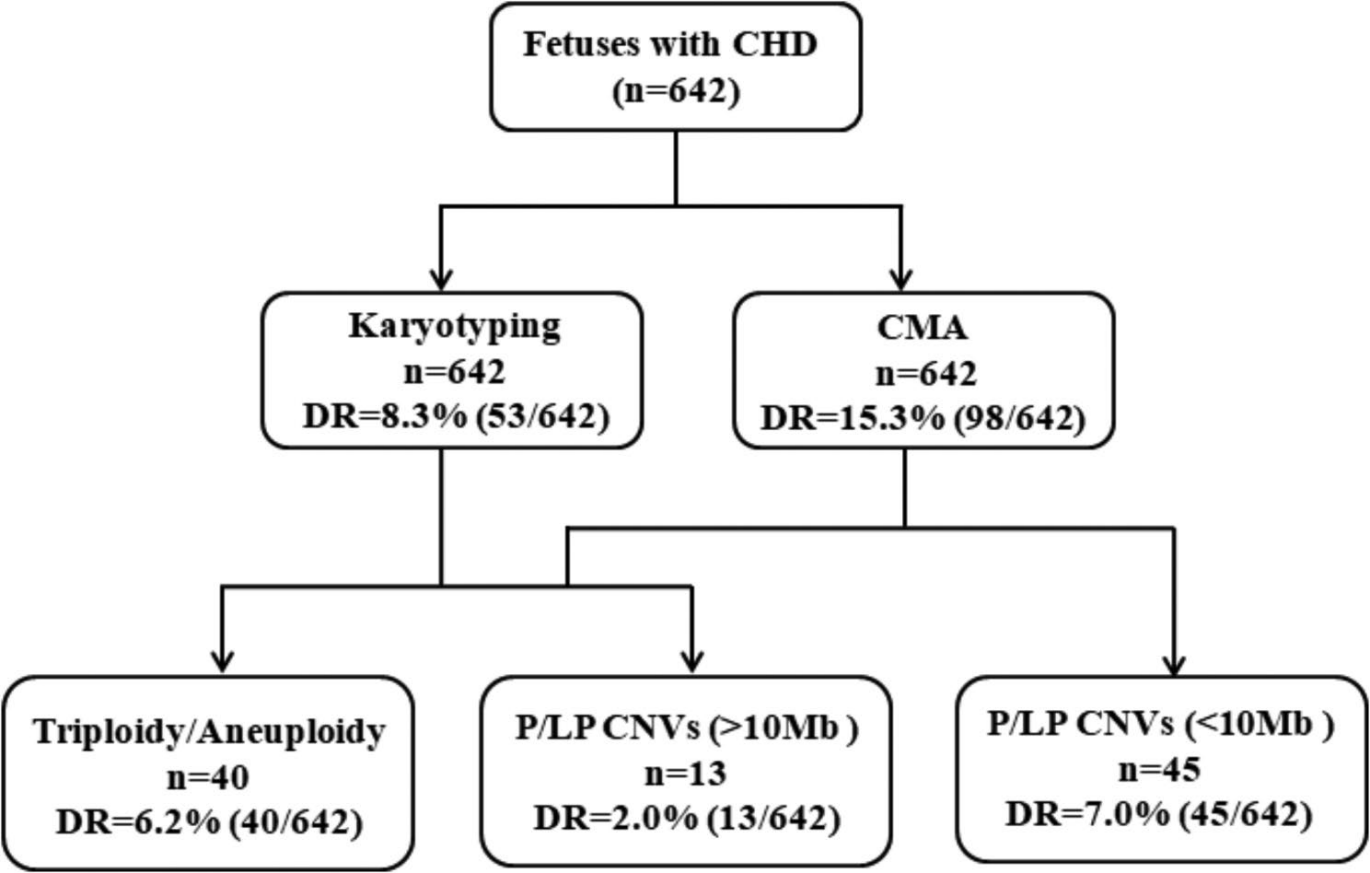
Flowchart of prenatal testing performed in 642 fetuses with CHD and the detection rate of karyotyping vs. CMA in this study. CHD: Congenital heart defects; CMA: chromosomal microarray analysis; CNVs: copy number variants; P/LP: pathogenic/likely pathogenic; DR: detection rate

A total of 642 fetuses with CHD underwent karyotyping and CMA. Chromosomal abnormalities were detected in 53 fetuses by conventional karyotyping, resulting in a detection rate of 8.3% (53/642). Meanwhile, CMA identified clinically significant chromosomal abnormalities in 98 fetuses, increasing the detection rate to 15.3% (98/642). Among them, 2 fetuses exhibited triploidy, 38 fetuses displayed aneuploidies, and 58 fetuses manifested pathogenic/likely pathogenic (P/LP) CNVs. Notably, compared to karyotyping, CMA not only identified all numerical chromosomal abnormalities and deletions/duplications detected by karyotyping but also revealed submicroscopic chromosomal abnormalities (Fig. 1).

### Chromosomal abnormalities in fetuses with CHD

After prenatal testing, numerical chromosomal abnormalities were detected in 40 (6.2%) fetuses, including 16 fetuses with trisomy 18 (T18), 15 fetuses with trisomy 21 (T21), 4 fetuses with trisomy 13 (T13), 3 fetuses with monosomy X (45, XO), 1 fetus with a karyotype of 69, XXX and another fetus with a karyotype of 69, XXY. Therefore, T18, T21 and T13 were the most prevalent numerical chromosomal abnormalities observed among fetuses with CHD in our study. Additionally, clinically significant CNVs were identified in a total of 58 (9.0%) fetuses, including 52 fetuses with pathogenic CNVs and 6 fetuses with likely pathogenic CNVs. Among these findings, the most common CNV was 22q11.2 deletion, accounting for 48.2% (28/58) of all the clinically significant CNVs detected. The other three recurrent CNV loci were 7q11.23 (involving *ELN*), 1q21.1q21.2 (involving *GJA5*) and 16p11.2 (involving *TXB6*). There were a total of 29 cases involving the 22q11.2 region (28 deletions and 1 duplication), 4 cases involving the 7q11.23 region (2 deletions and 2 duplications), 4 cases involving the 1q21.1q21.2 region (1 deletion and 3 duplications), and 2 cases involving the 16p11.2 region (1 deletion and 1duplication). The remaining clinically significant CNVs were detected only once, including 1q31.2q32.1 deletion (involving *CDC73*), 2q13 deletion (involving *BCL2L11)*, 2q37.1q37.3 deletion (involving *HDAC4*), 4p16.3q12 duplication, 4q32.1q35.2 duplication, 5q22.3q35.3 duplication (involving *NSD1*), 6q16.3q21 duplication, 6q25.3q27 deletion (involving *ARID1B* and *DLL1*), 8p23.3p23.1 deletion, 8p23.1p22 deletion (involving *GATA4*), 8q21.11q24.13 duplication, 8q24.3 duplication, 9p24.3p24.2 deletion, 9p24.3p13.1 duplication, 9q34.11q34.3 duplication, 9p24.2q13 duplication, 10q23.2q23.31 deletion (involving *PTEN*), 14q24.1q32.33 mosaic duplication, 16p13.12p13.11 deletion (involving *MYH11*), 17p12 duplication, 22q13.2q13.33 deletion (involving *TCF20*), Xp22.31q28 duplication and Xq22.2 duplication(involving *PLP1*). The cardiac ultrasound findings and pregnancy outcomes of cases with four recurrent CNV loci associated with CHD are summarized in Table 2. The details of 19 cases with CNVs detected only once are shown in supplement Table S1. Additionally, 32 (5.0%) fetuses were detected to harbor variants of uncertain significance (VUS), including 17 (2.6%) duplications, 9 (1.4%) deletions, and 6 (0.9%) regions of allelic homozygosity (ROHs). The details of these cases are listed in supplementary Table S2.

**Table 2:** The cardiac ultrasound findings and pregnancy outcomes of cases with four recurrent CNV loci associated with CHD

Region	Interval (Mb)	Size of CNVs (Mb)	CNVs type	Number of cases	Inheritance (*n*)	Disorder gene	Interpretation	Cardiac ultrasound findings (*n*)	Pregnancy outcomes (*n*)	Known syndrome related to CHD
22q11.2	18.6–21.8	1.28–3.16	Del	28	Unknown/Inherited/De novo (24/3/1)	*TBX1*	P	RAA (12), VSD (5), DORV (2), MCHA (2), TOF (1), IAA (1), HLHS (1), PA (1), PS (1), Unspecified (2)	TOP (25)/ Live birth (3)	DiGeorge syndrome
	18.6–21.4	2.95	Dup	1	Unknown	*TBX1*	P	PS(1)	TOP (1)	22q11.2 duplication syndrome
1q21.1q21.2	145.8-147.8	1.92	Del	1	De novo	*GJA5*	P	Unspecified (1)	TOP (1)	1q21.2 microdeletion syndrome
	146.1-147.8	1.65/1.71/1.73	Dup	3	Unknown/Inherited/De novo (1/1/1)	*GJA5*	P	MCHA (1), RAA (1), Unspecified (1)	TOP (2)/ Live birth (1)	1q21.1 duplication syndrome
7q11.23	72.6–74.1	1.42/1.51	Del	2	Unknown	*ELN*	P	MCHA (1), HLHS (1)	TOP (2)	Williams-Beuren syndrome
	72.6–74.1	1.48/2.08	Dup	2	Unknown	*ELN*	P	MCHA (1), PS (1)	TOP (2)	7q11.23 duplication syndrome
16p11.2	29.4–30.1	0.76	Del	1	Unknown	*TXB6*	P	MCHA (1)	TOP (1)	16p11.2 microdeletion syndrome
	29.5–30.3	0.74	Dup	1	Unknown	*TXB6*	P	RAA (1)	TOP (1)	16p11.2 duplication syndrome

CHD: Congenital heart defects; Mb: megabase; CNVs: copy number variants; Dup: duplication; Del: deletion; n: number of cases; P: pathogenic; RAA: Right aortic arch; VSD: Ventricular septal defect; DORV: Double outlet right ventricle; MCHA: Multiple complex heart anomalies; TOF: Tetralogy of fallot; IAA: Interrupted aortic arch; HLHS: Hypoplastic left heart syndrome; PA: Pulmonic atresia; PS: Pulmonic stenosis; TOP: termination of pregnancy

### Subgroup analysis of different types of CHD

According to prenatal ultrasound findings and Botto’s anatomical classification [14], all cases were categorized into 10 groups and 26 subgroups. Among them, ventricular septal defects (VSD) accounted for the most common heart defects, representing 44.5% (286/642), followed by right aortic arch (RAA) at 19.5%, multiple complex heart anomalies at 6.2%, coarctation of the aorta at 3.0%, pulmonic stenosis at 2.2%, and tetralogy of Fallot at 2.2%. The diagnostic yields were high in complex CHD (34.9%), conotruncal defects (28.6%), right ventricular outflow tract obstructive defects (RVOTO) (25.9%), atrioventricular septal defects (AVSD) (25.0%), and left ventricular outflow tract obstructive defects (LVOTO) (24.1%). On the other hand, the diagnostic yields were relatively low in other CHD (10.6%) and septal defects (10.9%). The types of CHD and CMA diagnostic yield of fetuses with CHD in this cohort are summarized in Table 3 and the distribution of chromosomal abnormalities in different CHD subgroups are listed in Table 4.

**Table 3:** Types of CHD and CMA diagnostic yield of fetuses with CHD in this cohort

Types	Number of fetuses (*N*)	Fraction (*N*/642)	Isolated CHD *n* (%)	Non-isolated CHD *n* (%)	DR (%)
**Septal defects**	**293**	**45.6%**	**223(76.1%)**	**70(23.9%)**	**32(10.9%)**
ASD	6	0.9%	3(50.0%)	3(50.0%)	2(33.3%)
VSD	286	44.5%	219(76.6%)	67(23.4%)	30(10.5%)
ASD + VSD	1	0.2%	1(100.0%)	0(0.0%)	0(0.0%)
**Conotruncal defects**	**35**	**5.5%**	**26(74.3%)**	**9(25.7%)**	**10(28.6%)**
d-TGA	6	0.9%	6(50.0%)	0(0.0%)	1(16.7%)
DORV	8	1.3%	5(62.5%)	3(37.5%)	4(50.0%)
Truncus arteriosus	5	0.8%	3(60.0%)	2(40.0%)	2(40.0%)
Interrupted aortic arch	2	0.3%	1(50.0%)	1(50.0%)	1(50.0%)
Tetralogy of Fallot	14	2.2%	11(78.6%)	3(21.4%)	2(14.3%)
**LVOTO**	**29**	**4.5%**	**21(72.4%)**	**8(27.6%)**	**7(24.1%)**
Aortic stenosis	3	0.5%	2(66.7%)	1(33.3%)	1(33.3%)
Coarctation of the aorta	19	3.0%	16(84.2%)	3(15.8%)	1(5.3%)
HLHS	7	1.1%	3(42.9%)	4(57.1%)	5(71.4%)
**RVOTO**	**27**	**4.2%**	**20(74.1%)**	**7(25.9%)**	**7(25.9%)**
Tricuspid atresia	1	0.2%	0(0.0%)	1(100.0%)	1(100.0%)
Pulmonic atresia	1	0.2%	1(100.0%)	0(0.0%)	1(100.0%)
Pulmonic stenosis	16	2.5%	12(75.0%)	4(25.0%)	2(12.5%)
Pulm valve stenosis	7	1.1%	5(71.4%)	2(28.6%)	3(42.9%)
Ebstein	2	0.3%	2(100.0%)	0(0.0%)	0(0.0%)
**AVSD**	**4**	**0.6%**	**2(50.0%)**	**2(50.0%)**	**1(25.0%)**
**APVR**	**1**	**0.2%**	**1(100.0%)**	0(0.0%)	0(0.0%)
**Heterotaxy**	**13**	**2.0%**	**12(92.3%)**	**1(7.7%)**	0(0.0%)
**Complex CHD**	**43**	**6.7%**	**19(44.2%)**	**24(55.8%)**	**15(34.9%)**
Multiple complex heart anomalies	40	6.2%	16(40.0%)	24(60.0%)	15(37.5%)
Single ventricle	2	0.3%	2(100.0%)	0(0.0%)	0(0.0%)
L-TGA	1	0.2%	1(100.0%)	0(0.0%)	0(0.0%)
**Other CHD**	**141**	**22.0%**	**124(87.9%)**	**17(12.1%)**	**15(10.6%)**
Right aortic arch	125	19.5%	111(88.8%)	14(11.2%)	14(11.2%)
Double aortic arch	6	0.9%	4(66.7%)	2(33.3%)	0(0.0%)
Vascular ring	10	1.6%	9(90.0%)	1(10.0%)	1(10.0%)
**Unspecified**	**56**	**8.7%**	**46(82.1%)**	**10(17.9%)**	**11(19.6%)**
**Total**	**642**	**100.0%**	**494(77.0%)**	**148(23.1%)**	**98(15.3%)**

CHD: Congenital heart defects; ASD: Atrial septal defect; VSD: Ventricular septal defect; d-TGA: d-Transposition of the great arteries; DORV: Double outlet right ventricle; LVOTO: Left ventricular outflow tract obstructive defects; HLHS: Hypoplastic left heart syndrome; RVOTO: Right ventricular outflow tract obstructive defects; AVSD: Atrioventricular septal defects; APVR: Anomalous pulmonary venous return; L-TGA: L-Transposition of the great arteries; n: number of cases; DR: detection rate

**Table 4:** Distribution and detection rates of chromosomal abnormalities in different CHD subgroups

CHD classfication	Total					Isolated CHD					Non-isolated CHD				
	*n*	DR (%)	NCA	*P*/LP CNVs	VUS	*n*	DR (%)	NCA	*P*/LP CNVs	VUS	*n*	DR (%)	NCA	*P*/LP CNVs	VUS
**Septal defects**	293	10.9%	21	11	13	223	6.7%	6	9	9	70	24.3%	15	2	4
ASD	6	33.3%	2	0	0	3	33.3%	1	0	0	3	33.3%	1	0	0
VSD	286	10.5%	19	11	13	219	6.4%	5	9	9	67	23.9%	14	2	4
ASD + VSD	1	0.0%	0	0	0	1	0.0%	0	0	0	0	0.0%	0	0	0
**Conotruncal defects**	35	28.6%	3	7	2	26	11.5%	1	2	1	9	77.8%	2	5	1
d-TGA	6	16.7%	0	1	1	6	16.7%	0	1	1	0	0.0%	0	0	0
DORV	8	50.0%	2	2	0	5	40.0%	1	1	0	3	66.7%	1	1	0
Truncus arteriosus	5	40.0%	1	1	0	3	0.0%	0	0	0	2	100.0%	1	1	0
Interrupted aortic arch	2	50.0%	0	1	0	1	0.0%	0	0	0	1	100.0%	0	1	0
Tetralogy of Fallot	14	14.3%	0	2	1	11	0.0%	0	0	0	3	66.7%	0	2	1
**LVOTO**	29	24.1%	3	4	4	21	9.5%	1	1	3	8	62.5%	2	3	1
Aortic stenosis	3	33.3%	0	1	0	2	0.0%	0	0	0	1	100.0%	0	1	0
Coarctation of the aorta	19	5.3%	0	1	3	16	0.0%	0	0	3	3	33.3%	0	1	0
HLHS	7	71.4%	3	2	1	3	66.7%	1	1	0	4	75.0%	2	1	1
**RVOTO**	27	25.9%	1	6	1	20	20.0%	0	4	0	7	42.9%	1	2	1
Tricuspid atresia	1	100.0%	1	0	0	0	0.0%	0	0	0	1	100.0%	1	0	0
Pulmonic atresia	1	100.0%	0	1	0	1	100.0%	0	1	0	0	0.0%	0	0	0
Pulmonic stenosis	16	12.5%	0	2	0	12	8.3%	0	1	0	4	25.0%	0	1	0
Pulm valve stenosis	7	42.9%	0	3	1	5	40.0%	0	2	0	2	60.0%	0	1	1
Ebstein	2	0.0%	0	0	0	2	0.0%	0	0	0	0	0.0%	0	0	0
**AVSD**	4	25.0%	1	0	0	2	0.0%	0	0	0	2	50.0%	1	0	0
**APVR**	1	0.0%	0	0	0	1	0.0%	0	0	0	0	0.0%	0	0	0
**Heterotaxy**	13	0.0%	0	0	0	12	0.0%	0	0	0	1	0.0%	0	0	0
**Complex CHD**	43	34.9%	5	10	3	19	26.3%	0	5	2	24	41.7%	5	5	1
Multiple complex heart anomalies	40	37.5%	5	10	2	16	31.3%	0	5	1	24	41.7%	5	5	1
Single ventricle	2	0.0%	0	0	0	2	0.0%	0	0	0	0	0.0%	0	0	0
L-TGA	1	0.0%	0	0	1	1	0.0%	0	0	1	0	0.0%	0	0	0
**Other CHD**	141	10.6%	1	14	4	124	9.7%	0	12	4	17	17.7%	1	2	0
Right aortic arch	125	11.2%	0	14	3	111	10.8%	0	12	3	14	14.3%	0	2	0
Double aortic arch	6	0.0%	0	0	0	4	0.0%	0	0	0	2	0.0%	0	0	0
Vascular ring	10	10.0%	1	0	1	9	0.0%	0	0	1	1	100.0%	1	0	0
**Unspecified**	56	19.6%	5	6	5	46	17.4%	2	6	3	10	30.0%	3	0	2
**Total**	642	15.3%	40	58	32	494	9.9%	10	39	22	148	33.1%	30	19	10

CHD: Congenital heart defects; ASD: Atrial septal defect; VSD: Ventricular septal defect; d-TGA: d-Transposition of the great arteries; DORV: Double outlet right ventricle; LVOTO: Left ventricular outflow tract obstructive defects; HLHS: Hypoplastic left heart syndrome; RVOTO: Right ventricular outflow tract obstructive defects; AVSD: Atrioventricular septal defects; APVR: Anomalous pulmonary venous return; L-TGA: L-Transposition of the great arteries; n: number of cases; DR: detection rate; NCA: numerical chromosomal abnormalities; P/LP: pathogenic/likely pathogenic; CNVs: copy number variants; VUS: variants of uncertain significance

### Diagnostic yield of CMA in isolated vs. non-isolated CHD

In this study, a total of 494 cases presenting solely with cardiac ultrasound anomalies were classified as the isolated CHD group; meanwhile, 148 cases accompanied by extracardiac ultrasound anomalies were classified as the non-isolated CHD group. Extracardiac ultrasound anomalies included soft markers (*n* = 73), additional structural anomalies (*n* = 61), intrauterine growth retardation (IUGR) (*n* = 7), and polyhydramnios (*n* = 7). The frequencies of clinically significant chromosomal abnormalities were found to be 9.9% (49/494), 35.6% (26/73), 36.1% (22/61), 14.3% (1/7) and 0.0% (0/7) in fetuses with isolated CHD, CHD with soft markers, CHD with additional structural anomalies, CHD with IUGR, and CHD with polyhydramnios, respectively. Compared to the isolated CHD group, the detection rate of clinically significant chromosomal abnormalities was significantly higher in the CHD with soft markers group (35.6% vs. 9.9%, *P* < 0.0001) and the CHD with additional structural anomalies group (36.1% vs. 9.9%, *P* < 0.0001); however, no highly significant differences were observed between these two groups (35.6% vs. 36.1%, *P* = 0.956). The CHD group with soft markers consisted of 34 cases of VSD with soft markers and 39 cases of other cardiac malformations with soft markers. The detection rate of clinically significant chromosomal abnormalities in the group with other cardiac malformations and soft markers was higher than that in the group with VSD and soft markers, but the difference was not statistically significant (43.6% vs. 26.5%, *P* = 0.148). However, compared to the isolated VSD group, the detection rate was significantly higher in the VSD group with soft markers (26.5% vs. 6.4%, *P* < 0.05). In the CHD with soft markers group, there were 73 fetuses with a single soft marker and 3 fetuses with multiple soft markers. The detection rate of clinically significant chromosomal abnormalities in fetuses with a single soft marker was lower than that in fetuses with multiple soft markers, but this difference was not statistically significant (34.3% vs. 66.7%, *P* = 0.287). Among this group, the detection rates of clinically significant chromosomal abnormalities were high in fetuses exhibiting increased nuchal translucency (4/4, 100.0%), pyelectasis (1/1, 100.0%), mild ventriculomegaly (3/4, 75.0%), and absent or shortened nasal bone (12/19, 63.2%). Similarly, in the CHD with additional structural anomalies group, 40 fetuses had a single structural anomaly and 21 fetuses had multiple structural anomalies. The detection rate of clinically significant chromosomal abnormalities in fetuses with multiple structural anomalies was higher than that in fetuses with a single structural anomaly; however, there were no statistically significant differences (52.4% vs. 27.5%, *P* = 0.091). The detection rates of clinically significant chromosomal abnormalities were high in fetuses with exomphalos (3/3, 100.0%), diaphragmatic hernia (1/1, 100.0%), nuchal cystic hygroma (1/2, 50.0%), urinary tract system anomalies (3/9, 33.3%), and facial abnormalities (2/6, 33.3%) within this group. The detection rates of chromosomal abnormalities in fetuses with isolated CHD and non-isolated CHD are presented in Table 5.

**Table 5:** Detection rates of chromosomal abnormalities in fetuses with isolated CHD and non-isolated CHD

CHD classifications	*n*	DR (%)	NCA	*P*/LP CNVs	VUS
**Isolated CHD**	**494**	**9.9%**	**10**	**39**	**22**
**Non-isolated CHD**	**148**	**33.1%**	**30**	**19**	**10**
**CHD with soft markers**	**73**	**35.6%**	**16**	**10**	**5**
CHD with single soft marker	70	34.3%	15	9	5
Single umbilical artery	21	9.5%	1	1	1
Absent or shortened nasal bone	19	63.2%	8	4	2
Echogenic bowel	8	12.5%	0	1	1
Choroid plexus cysts	7	0.0%	0	0	1
Mild ventriculomegaly	4	75.0%	1	2	0
Increased nuchal translucency	4	100.0%	4	0	0
Enlarged cisterna magna	4	25.0%	1	0	0
Thickened nuchal fold	1	0.0%	0	0	0
Persistent right umbilical vein	1	0.0%	0	0	0
Pyelectasis	1	100.0%	0	1	0
CHD with multiple soft markers	3	66.7%	1	1	0
**CHD with additional structural anomalies**	**61**	**36.1%**	**14**	**8**	**3**
CHD with single structural anomaly	40	27.5%	6	5	2
Urinary tract system	9	33.3%	1	2	1
Central nervous system	8	12.5%	0	1	1
Facial abnormalities	6	33.3%	1	1	0
Gastrointestinal system	4	0.0%	0	0	0
Respiratory system	5	0.0%	0	0	0
Exomphalos	3	100.0%	3	0	0
Nuchal cystic hygroma	2	50.0%	1	0	0
Placenta	2	0.0%	0	0	0
Diaphragmatic hernia	1	100.0%	0	1	0
CHD with multiple structural anomalies	21	52.4%	8	3	1
**CHD with IUGR**	**7**	**14.3%**	**0**	**1**	**1**
**CHD with polyhydramnios**	**7**	**0.0%**	**0**	**0**	**1**

CHD: Congenital heart disease; n: number of cases; DR: detection rate; NCA: numerical chromosomal abnormalities; P/LP: pathogenic/likely pathogenic; CNVs: copy number variants; VUS: variants of uncertain significance; IUGR: intrauterine growth retardation

The overall detection rate of clinically significant chromosomal abnormalities was significantly higher in the non-isolated group compared to the isolated group (33.1% vs. 9.9%, *P* < 0.0001). Additionally, within the CHD subgroups, there was a significantly higher detection rate observed in the non-isolated VSD group compared to the isolated VSD group (23.9% vs. 6.4%, *P* < 0.0001). Interestingly, numerical chromosomal abnormalities were more likely to occur in the non-isolated group than the isolated group (20.3% vs. 2.0%, *P* < 0.0001). However, there were no significant differences in the incidence of pathogenic/likely pathogenic CNVs (12.8% vs. 7.9%, *P* = 0.065) and VUS (6.8% vs. 4.5%, *P* = 0.258) between these two groups. The comparison of the distribution of chromosomal abnormalities between different CHD groups is summarized in Table 6.

**Table 6:** Comparison of the distribution of chromosomal abnormalities between different CHD groups

CHD groups	NCA	*P*/LP CNVs	Clinically significant chromosomal abnormalities	VUS
Isolated CHD group vs. non-isolated CHD group *P* value	< 0.0001	0.065	< 0.0001	0.258
Isolated VSD group vs. non-isolated VSD group *P* value	< 0.0001	> 0.9999	< 0.0001	0.510
CHD with soft markers group vs. isolated CHD group *P* value	< 0.0001	0.116	< 0.0001	0.374
CHD with additional structural anomalies group vs. isolated CHD group *P* value	< 0.0001	0.167	< 0.0001	0.748
VSD with soft markers group vs. isolated VSD group P value	< 0.0001	> 0.9999	< 0.005	0.646
CHD with soft markers group vs. CHD with additional structural anomalies group *P* value	0.886	0.921	0.956	0.727
CHD with single soft marker group vs. CHD with multiple soft markers group *P* value	0.529	0.361	0.287	> 0.9999
CHD with single structural anomaly group vs. CHD with multiple structural anomalies group P value	0.057	> 0.9999	0.091	> 0.9999

CHD: Congenital heart disease; VSD: Ventricular septal defect; NCA: numerical chromosomal abnormalities; P/LP: pathogenic/likely pathogenic; CNVs: copy number variants; VUS: variants of uncertain significance

## Discussion

In this study, we conducted a cohort study on fetuses with various types of CHD to evaluate the prevalence of chromosomal abnormalities and the clinical utility of CMA. Our results demonstrated a significant increase in diagnostic yield through CMA testing among fetuses with CHD. The overall detection rate achieved by CMA was 15.3%, which represents a 7% incremental diagnostic yield over karyotype analysis, consistent with several previous studies [9, 16]. In recent years, CMA has been widely used in prenatal diagnosis, including fetal cardiac ultrasound anomalies. Numerous studies have reported varying detection rates ranging from 10.1 to 24.5% when using CMA for fetal CHD diagnosis [16–22]. However, in comparison to previous studies [16–22], our cohort exhibited relatively lower rates of diagnosing chromosomal abnormalities, particularly aneuploidies. The observed discrepancy can be attributed to several factors, including potential biases in case selection, differences in the proportion of CHD subgroups, and patients’ preference for noninvasive prenatal strategies. Nevertheless, it is indisputable that CMA should be recommended as a first-tier diagnostic technique for prenatal cases of CHD.

As the first recognized genetic cause of CHD, aneuploidy accounts for approximately 14% of all genetic causes of syndromic CHD [2, 23]. T18 was identified as the most prevalent form of aneuploidy detected in fetuses with CHD in this study, followed by T21 and T13. Additionally, CNVs have emerged as significant genetic contributors to CHD in recent years [24–26]. In our study, a total of fifty-two pathogenic CNVs and six likely pathogenic CNVs were identified. The most common CNV was the 22q11.2 deletion, accounting for 50.0% of all clinically significant CNVs detected. Other recurrent CNV loci included 7q11.23 (involving *ELN*), 1q21.1q21.2 (involving *GJA5*) and 16p11.2 (involving *TXB6*). These four recurrent CNV loci have previously been reported to be associated with CHD; however, patients with these CNVs exhibit highly variable clinical phenotypes [27–32]. Moreover, nineteen rare CNVs were detected only once in our study, which might either be the genetic cause for CHD or a secondary finding. Nevertheless, their relationship to prenatal ultrasound phenotype remains unclear and requires further confirmation. Given that a large portion of cardiovascular anomalies in fetuses with CHD are undetectable even with advanced ultrasound equipment and well-trained sonographers [33], it is extremely challenging for clinicians to accurately assess their true condition based solely on ultrasound findings. Therefore, precise genetic diagnosis is crucial for prenatal genetic counseling and prognosis evaluation of cases with CHD. Furthermore, this study identified thirty-two variants of uncertain significance, which posed great challenges to clinicians during genetic counseling due to the ambiguity surrounding their association with clinical phenotypes. Through postnatal follow-up analysis, we observed that a large proportion of participants whose fetuses were diagnosed with VUS chose to terminate their pregnancies, especially when the source of these variants was not confirmed. It was undeniable that the genetic diagnosis of fetuses with CHD had some influence on the outcome of their pregnancies, so clinicians should exercise extreme caution in genetic counseling for cases involving VUS.

Our study revealed significant differences in the overall detection rate of clinically significant chromosomal abnormalities between the non-isolated CHD group and the isolated CHD group (33.1% vs. 9.9%, *P* < 0.0001), which is consistent with findings from several previous studies [16, 17, 19, 21]. Among subgroups of CHD, fetuses with complex CHD had the highest detection rate at 34.9%, followed by conotruncal defects at 28.6%, RVOTO at 25.9%, AVSD at 25.0%, and LVOTO at 24.1%. The detection rate for fetuses with VSD was relatively low, only reaching 10.1%; however, within the VSD subgroup, non-isolated cases had a significantly higher detection rate compared to isolated cases (23.9% vs. 6.4%, *P* < 0.0001). Maya et al. found that the risk of chromosomal abnormalities in isolated VSD showed no difference from the background risk, but the risk was higher in non-isolated VSD [34], which is consistent with our findings. Several recent studies have reported that the risk of chromosomal abnormalities in pregnancies with isolated VSD is not significant [17, 34, 35], thus it is considered unreasonable to perform invasive prenatal testing for isolated VSD. To better guide clinical decision-making in cases of prenatal isolated VSD, the correlation between chromosomal abnormalities and isolated VSD are yet to be further investigated.

In line with previous studies [16, 19–21, 36], our study discovered that the detection rate in the group of CHD with additional structural anomalies was significantly higher than that in the isolated CHD group (36.1% vs. 9.9%, *P* < 0.0001). However, the comparison of detection rates between the group of CHD with soft markers and the isolated CHD group was controversial. Wang et al. found no significant differences in the detection rates between these two groups (19.8% vs. 14.3%, ns) [16], while our study observed significant differences (35.6% vs. 9.9%, *P* < 0.0001). These inconsistent findings could be partially explained by differences in cohort size, inclusion criteria, and proportion of CHD subgroups. The soft markers, although they may be transient and self-resolve later in pregnancy, are acknowledged as underlying risk factors for fetal aneuploidy [37]. Multiple studies have reported that the risk of chromosomal abnormalities increases when multiple soft markers appear or when soft markers are combined with structural abnormalities [37–40]. Therefore, we suggest paying special attention to prenatal cases of CHD combined with soft markers, as well as cases of CHD combined with additional structural anomalies.

The remarkable strength of this study lies in the large size of our cohort from a single center, which enabled us to compare the detection rate of chromosomal abnormalities among different CHD subgroups and investigate the correlation between chromosomal abnormalities and prenatal cardiac ultrasound phenotypes. However, our study had several limitations. These included the potential selection bias caused by patients having to pay for invasive prenatal testing and some patients refusing further testing. Additionally, CMA could not detect sequence variants; moreover, most patients declined subsequent whole-exome sequencing which resulted in incomplete genetic diagnoses. Finally, although our cohort was large enough overall, the small sample sizes in certain CHD subgroups limited the analysis.

## Conclusions

We conducted one of the largest cohort studies to assess the clinical value of CMA in prenatal genetic diagnoses of fetuses with CHD from a single center. CMA, being a reliable and high-resolution technique, should be recommended as the front-line test for prenatal diagnosis of fetuses with CHD. Our findings revealed that the prevalence of chromosomal abnormalities varied greatly among different subgroups of CHD, and special attention should be given to prenatal non-isolated cases of CHD, especially those accompanied by additional structural anomalies or soft markers.

### Electronic supplementary material

Below is the link to the electronic supplementary material.

Supplementary Material 1

Supplementary Material 2

## Data Availability

All relevant data generated or analyzed during this study are included in this published article. If further datasets are requested, these are available on request from the corresponding author.

## Abbreviations

CHD: Congenital heart defects
CMA: Chromosomal microarray analysis
CNVs: Copy number variants
P: Pathogenic
LP: Likely pathogenic
VUS: Variants of uncertain significance
ASD: Atrial septal defect
VSD: Ventricular septal defect
d-TGA: d-Transposition of the great arteries
DORV: Double outlet right ventricle
LVOTO: Left ventricular outflow tract obstructive defects
HLHS: Hypoplastic left heart syndrome
RVOTO: Right ventricular outflow tract obstructive defects
AVSD: Atrioventricular septal defects
APVR: Anomalous pulmonary venous return
L-TGA: L-Transposition of the great arteries
TOP: Termination of pregnancy
