# Supplementary material for: Prenatal chromosomal microarray analysis in a large Chinese cohort of fetuses with congenital heart defects: a single center study
Source: Orphanet J Rare Dis. 2024 Aug 22;19:307. doi: 10.1186/s13023-024-03317-4 (PMC11342572; doi:10.1186/s13023-024-03317-4)
Supplement: Supplementary file 1 — Supplementary Material 1 [file 13023_2024_3317_MOESM1_ESM.docx]

**Table S1-**The details of 19 cases with pathogenic / likely pathogenic CNVs detected only once.

| Case | CNVs: position(GRCh37) | Size of CNVs (Mb) | CNVs type | Cardiovascular ultrasound findings | Extracardiac ultrasound ﬁndings | Inheritance | Interpretation | Pregnancy outcomes |
| --- | --- | --- | --- | --- | --- | --- | --- | --- |
| 1 | arr 1q31.2q32.1(192146049_201855150)×1 | 9.70Mb | Del | VSD | Absent nasal bone | de novo | P | TOP |
| 2 | arr 2q37.1q37.3(234180469_242782258)×1 | 8.60Mb | Del | VSD | Multiple structural anomalies | de novo | P | TOP |
| 3 | arr 2q13(111382573_113111856)×1 | 1.72Mb | Del | VSD | - | Maternal  inherited | LP | Live birth |
| 4 | arr 4p16.3q12(68345_56302561)×3 | 56.23Mb | Dup | Multiple complex heart anomalies | - | de novo | P | TOP |
| 5 | arr 4q32.1q35.2(156228522_190957460)×3 | 34.72Mb | Dup | VSD | - | de novo | P | TOP |
| 6 | arr 5q22.3q35.3(113201524_180677927)×3 | 67.41Mb | Dup | Multiple complex heart anomalies | Urinary tract system | de novo | P | TOP |
|  | arr Xp22.31q28(8754814_155233098)×3 | 146.47Mb | Dup |  |  |  | P |  |
| 7 | arr 6q16.3q21(104495018_112902562)×3 | 8.40Mb | Dup | VSD | - | NA | LP | TOP |
| 8 | arr 6q25.3q27(157075546_170914297)×1 | 13.83Mb | Del | Multiple complex heart anomalies | - | de novo | P | TOP |
| 9 | arr 8p23.1p22(11583624_12986826)×1 | 1.40Mb | Del | PVS, PLSVC, Arrhythmia | - | NA | LP | TOP |
| 10 | arr 8p23.3p23.1(158049_8825096)×1 | 8.66Mb | Del | VSD, TA | Facial abnormalities | de novo | P | TOP |
|  | arr 8q21.11q24.13(74432203_126108281)×3 | 51.67Mb | Dup |  |  |  | P |  |
|  | arr 8q24.3(141076238_146295771)×3 | 5.22Mb | Dup |  |  |  | LP |  |
| 11 | arr 9p24.3p13.1(208455_38787480)×3 | 38.57Mb | Dup | CoA, HLHS, PLSVC | Absent nasal bone,  Mild ventriculomegaly | de novo | P | TOP |
| 12 | arr 9q34.11q34.3(133817639_141153431)×3 | 7.33Mb | Dup | HLHS | - | NA | LP | TOP |
| 13 | arr 9p24.2q13(2539221_68216577)×3 | 65.67Mb | Dup | Multiple complex heart anomalies | Mild ventriculomegaly | de novo | P | TOP |
| 14 | arr 10q23.2q23.31(89014064_89764071)×1 | 750kb | Del | PVS | - | Paternal  inherited | P | Live birth |
| 15 | arr 14q24.1q32.33(69136271_107284437)×2.29 | 38.14Mb | Mos Dup | TOF, RAA | Diaphragmatic hernia | de novo | LP | TOP |
| 16 | arr 16p13.12p13.11(14770632_16458424)×1 | 1.68Mb | Del | VSD | - | NA | P | Live birth |
| 17 | arr 17p12(14087918_15473312)×3 | 1.38Mb | Dup | VSD | Mild ventriculomegaly | Maternal inherited | P | Live birth |
| 18 | arr 22q13.2q13.33(41813913_50263068)×1 | 8.44Mb | Del | AS, VSD, Hydropericardium | Mild ventriculomegaly, Urinary tract system | de novo | P | TOP |
| 19 | arr Xq22.2(102769945_103223398)×4 | 453kb | Dup | d-TGA | - | NA | P | TOP |

CNVs: copy number variants; Mb: megabase; Dup: duplication; Mos Dup: mosaic duplication; Del: deletion; VSD: Ventricular septal defect; PVS: Pulm valve stenosis; PLSVC: Persistent left superior vena cava; TA: Truncus arteriosus; CoA: Coarctation of the aorta; HLHS: Hypoplastic left heart syndrome; TOF: Tetralogy of fallot; RAA: Right aortic arch; AS: Aortic stenosis; d-TGA: d-Transposition of the great arteries; NA: Not available; P: pathogenic; LP: likely pathogenic; TOP: termination of pregnancy; -: No exist.
