# Supplementary material for: Prenatal chromosomal microarray analysis in a large Chinese cohort of fetuses with congenital heart defects: a single center study
Source: Orphanet J Rare Dis. 2024 Aug 22;19:307. doi: 10.1186/s13023-024-03317-4 (PMC11342572; doi:10.1186/s13023-024-03317-4)
Supplement: Supplementary file 2 — Supplementary Material 2 [file 13023_2024_3317_MOESM2_ESM.docx]

**Table S2**- The details of 32 cases with variants of uncertain significance detected.

| Case | | CNVs: position(GRCh37) | Size of  CNVs (Mb) | CNVs type | Cardiovascular ultrasound findings | Extracardiac ultrasound ﬁndings | Inheritance | Interpretation | Pregnancy outcomes |
| --- | --- | --- | --- | --- | --- | --- | --- | --- | --- |
| 1 | arr 2p12(78784099_79973436)×3 | | 1.18Mb | Dup | VSD | - | NA | VUS | TOP |
| 2 | arr 2p12(78608100_80000425)×3 | | 1.39Mb | Dup | Unspecified | - | NA | VUS | TOP |
| 3 | arr 2q12.3q13(108513707_110442979)×3 | | 1.92Mb | Dup | Vascular ring | - | Paternal inherited | VUS | Live birth |
| 4 | arr 3p26.3(1448822_2533983)×3 | | 1.08Mb | Dup | AS | - | Maternal inherited | VUS | Live birth |
| 5 | arr 3p14.3(56428106_58123249)×3 | | 1.69Mb | Dup | pulmonic stenosis | Echogenic bowel | NA | VUS | TOP |
| 6 | arr 4q26q27(118361866_121564291)×3 | | 3.20Mb | Dup | VSD、CoA | - | Maternal inherited | VUS | Live birth |
| 7 | arr 4q35.2(189384162_190957460)×1 | | 1.53Mb | Del | TOF | IUGR、Polyhydramnios | NA | VUS | TOP |
| 8 | arr 4q13.1(63719086_65452238)×1 | | 1.73Mb | Del | Vascular ring | - | NA | VUS | TOP |
| 9 | arr 5q23.1(118507169_119850070)×3 | | 1.34Mb | Dup | VSD | - | Paternal inherited | VUS | Live birth |
| 10 | arr 5q35.3(177888405_178936592)×3 | | 1.04Mb | Dup | VSD | Choroid plexus cysts | Paternal inherited | VUS | Live birth |
| 11 | arr 6p23p22.3(14041412_15281568)×3 | | 1.24Mb | Dup | VSD | - | Maternal inherited | VUS | Live birth |
| 12 | arr 6q24.3q25.1(148689991_149893356)×1 | | 1.20Mb | Del | Unspecified | - | Maternal inherited | VUS | Live birth |
| 13 | arr 7q31.33(123782969 _125751364)×3 | | 1.96Mb | Dup | RAA | - | NA | VUS | Live birth |
| 14 | arr 7q35(146954513_147316326)×1 | | 362kb | Del | VSD | multiple structural anomalies | NA | VUS | TOP |
| 15 | arr 8p23.2(3688710_5950611)×3 | | 2.26Mb | Dup | VSD | - | Paternal inherited | VUS | Live birth |
| 16 | arr 10p15.1p14(66369608_7356251)×3 | | 986kb | Dup | VSD | - | Paternal inherited | VUS | Live birth |
| 17 | arr 10p13(12874177_13605956)×1 | | 731kb | Del | RAA | - | Maternal inherited | VUS | Live birth |
| 18 | arr 10q11.22(46293590_48167553)×3 | | 1.87Mb | Dup | HLHS | Absent nasal bone | NA | VUS | TOP |
| 19 | arr 13q33.1(103686191_104205800)×1 | | 520kb | Del | Multiple complex heart anomalies | - | NA | VUS | TOP |
| 20 | arr 15q13.3(32011458_32439300)×3 | | 428kb | Dup | VSD | - | Maternal inherited | VUS | Live birth |
| 21 | arr 15q11.2(22770421_23625785)×1 | | 855kb | Del | VSD | - | NA | VUS | TOP |
| 22 | arr 15q11.2(22770421_23662482)×1 | | 892kb | Del | VSD | Absent nasal bone | NA | VUS | TOP |
| 23 | arr 16p13.11(15058820_16538596)×3 | | 1.48Mb | Dup | d-TGA | - | NA | VUS | TOP |
| 24 | arr 17p13.3(2286098 _2546406)×3 | | 260kb | Dup | VSD | - | Paternal inherited | VUS | Live birth |
| 25 | arr 20q11.22q11.23(34160065_34897085)×3 | | 737kb | Dup | Multiple complex heart anomalies | Urinary tract system | NA | VUS | TOP |
| 26 | arr Xp22.31(6449752_8143509)×1 | | 1.69Mb | Del | Unspecified | Single umbilical artery | NA | VUS | TOP |
| 27 | arr 6q12q14.1(67025239_77481958)×2 hmz | | 10.45Mb | ROH | VSD | - | de novo | VUS | Live birth |
| 28 | arr 16q12.2q22.3(56006602_73365284)×2 hmz | | 17.35Mb | ROH | Unspecified | - | de novo | VUS | Live birth |
| 29 | arr 11p13p11.12(36066655_51550787)×2 hmz | | 15.48Mb | ROH | VSD | - | de novo | VUS | Live birth |
| 30 | arr 7p14.1q21.11(41665452_82756536)×2 hmz | | 41.09Mb | ROH | VSD | Polyhydramnios | de novo | VUS | Live birth |
| 31 | arr 5p15.3q35.3(113576_180692321)×2 hmz | | 180.57Mb | ROH | CoA | - | de novo | VUS | TOP |
| 32 | arr 8p23.3q24.3(168484_146292734)×2 hmz | | 146.12Mb | ROH | d-TGA | - | de novo | VUS | TOP |

CNVs: copy number variants; Mb: megabase; Dup: duplication; Del: deletion; ROH: region of allelic homozygosity; VSD: Ventricular septal defect; AS: Aortic stenosis; CoA: Coarctation of the aorta; TOF: Tetralogy of fallot; RAA: Right aortic arch; HLHS: Hypoplastic left heart syndrome; d-TGA: d-Transposition of the great arteries; IUGR:; NA: Not available; VUS: variants of uncertain significance; TOP: termination of pregnancy; -: No exist.
